# Supplementary figures and images for: Modulating myoblast differentiation with RNA-based controllers
Source: PLoS One. 2022 Sep 27;17(9):e0275298. doi: 10.1371/journal.pone.0275298 (PMC9514614; doi:10.1371/journal.pone.0275298)

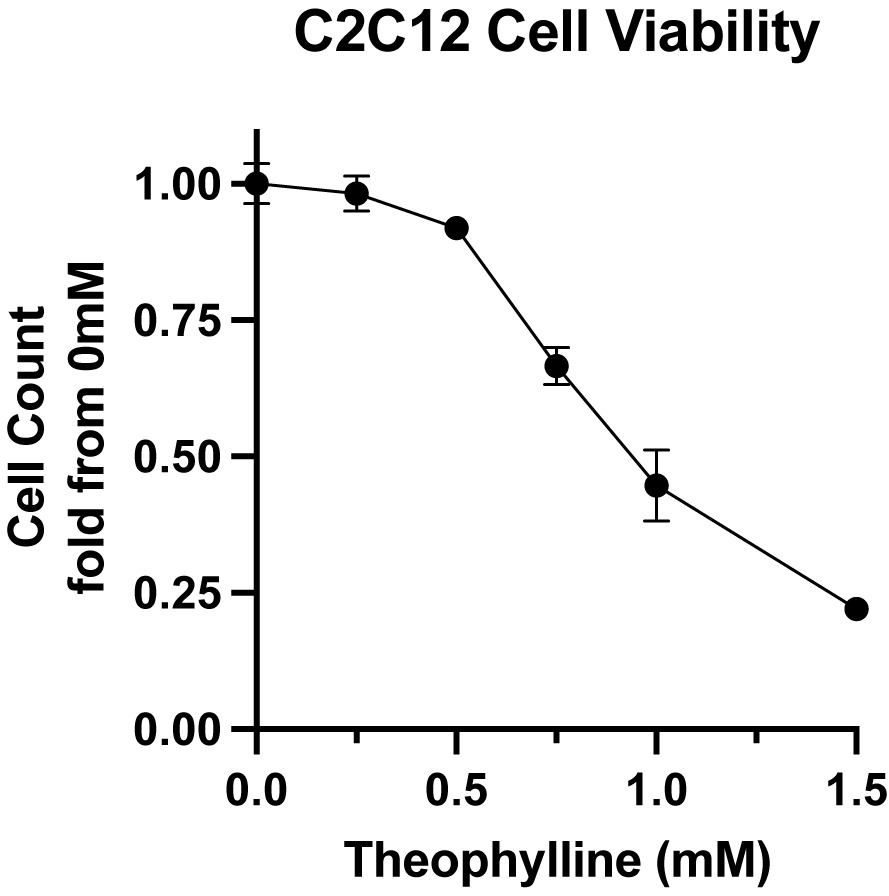

Supplement: S1 Fig — Dose response curve for C2C12 cell count showing relative cell count normalized to the cell count at 0 mM theophylline. Error bars indicate standard deviation of two biological replicates. (TIF) [file pone.0275298.s001.tif]

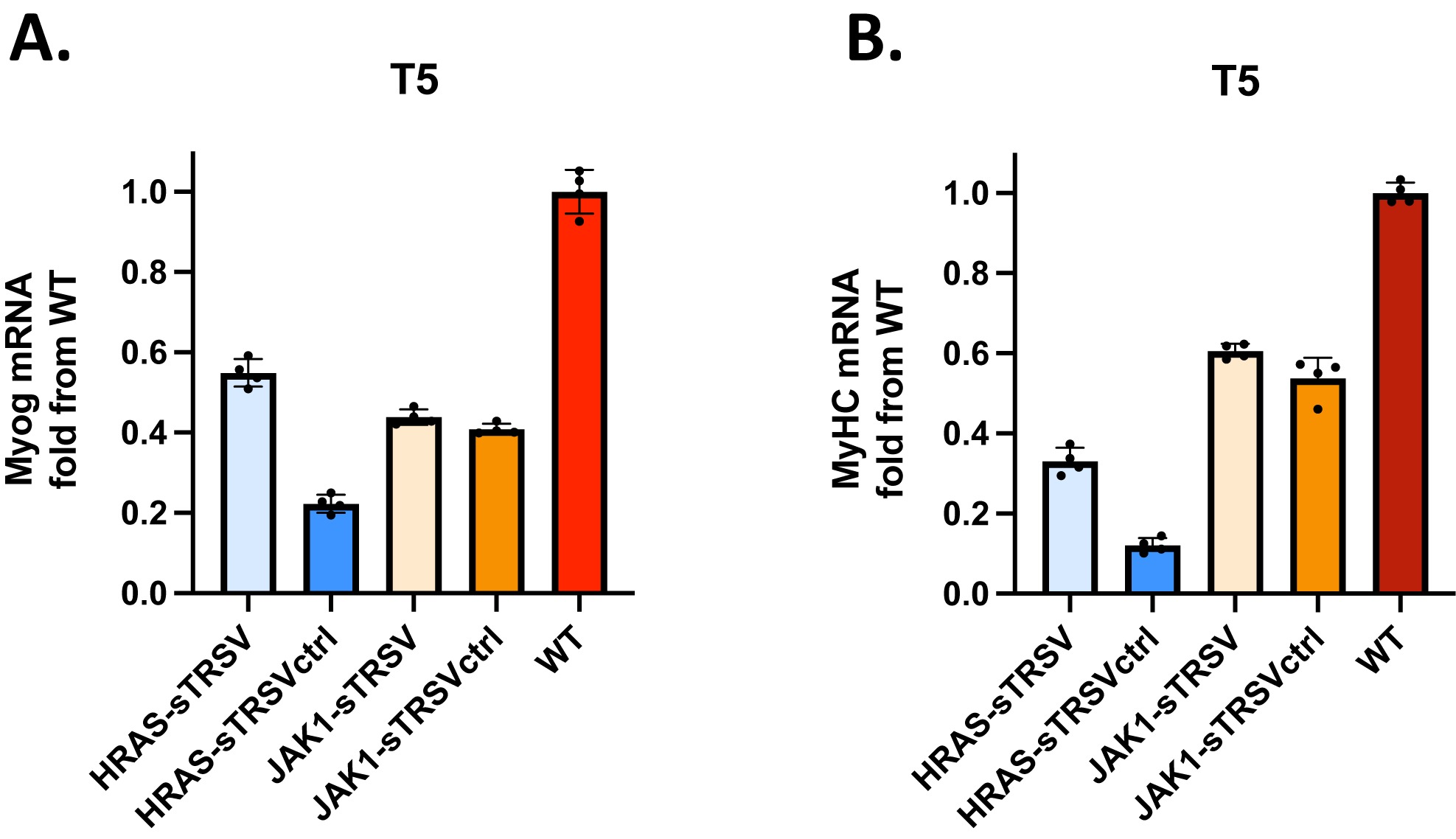

Supplement: S2 Fig — A. Real-time RT-qPCR detection of Myogenin five days after transient transfection of HRAS and JAK1 constructs compared to wild-type myoblasts. B. Real-time RT-qPCR detection of MyHC five days after transient transfection of HRAS and JAK1 constructs compared to wild-type myoblasts. Error bars indicate standard deviation of four biological replicates. (TIF) [file pone.0275298.s002.tif]

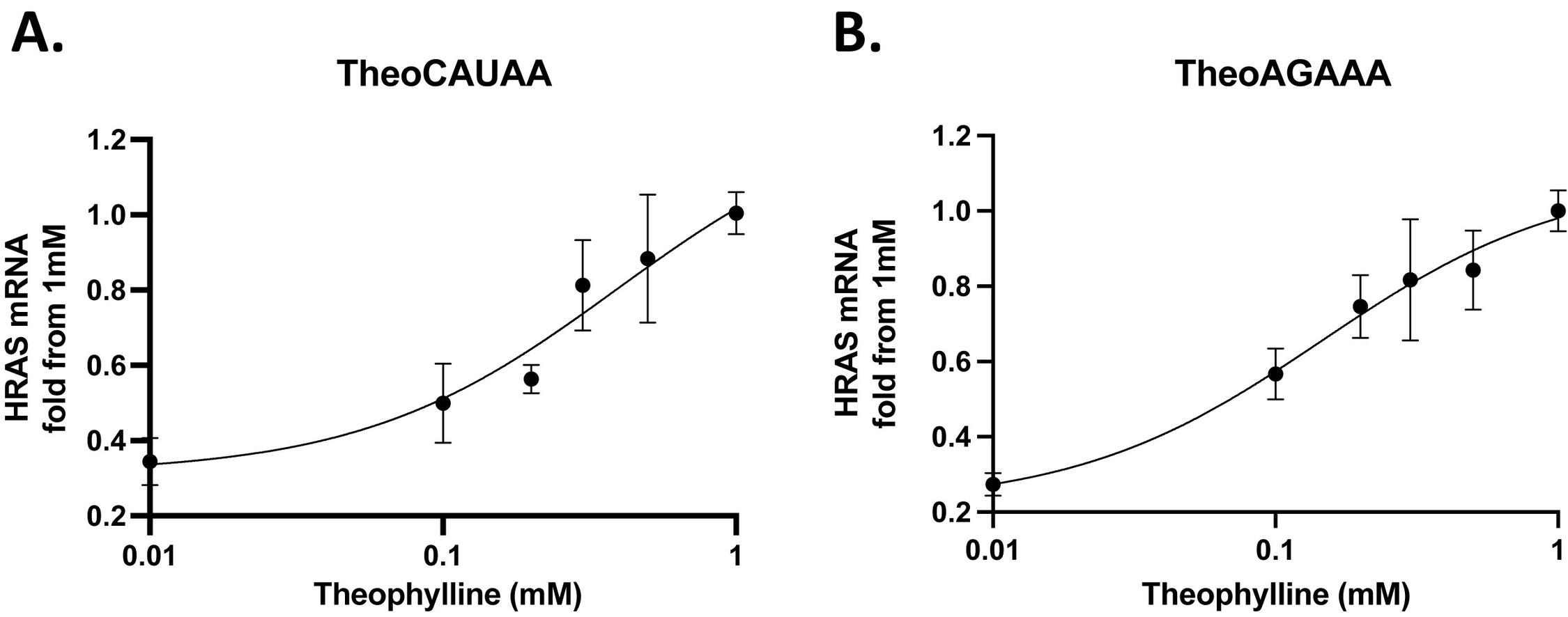

Supplement: S3 Fig — A. Dose response curve for TheoCAUAA ribozyme switch showing relative HRAS mRNA transcript levels normalized to transcript levels at 1 mM theophylline. B. Dose response curve for TheoAGAAA ribozyme switch showing relative HRAS mRNA transcript levels normalized to transcript levels at 1 mM theophylline. Error bars indicate standard deviation of three or more biological replicates. (TIF) [file pone.0275298.s003.tif]

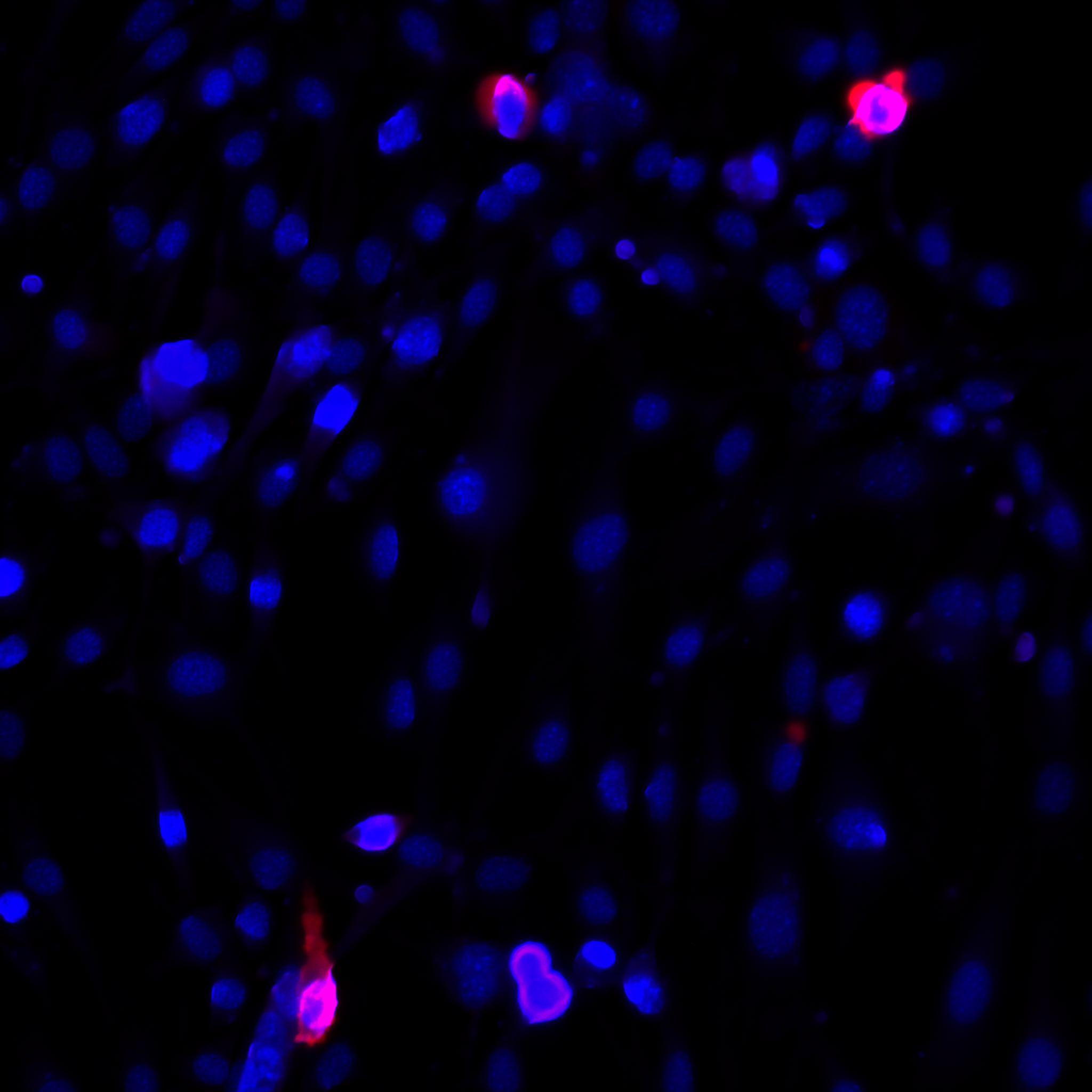

Supplement: S1 File — (ZIP) [file pone.0275298.s004.zip › Supporting Information (images)/Fig5A-08-1mM-HRAS-sTRSVctrl.tif]

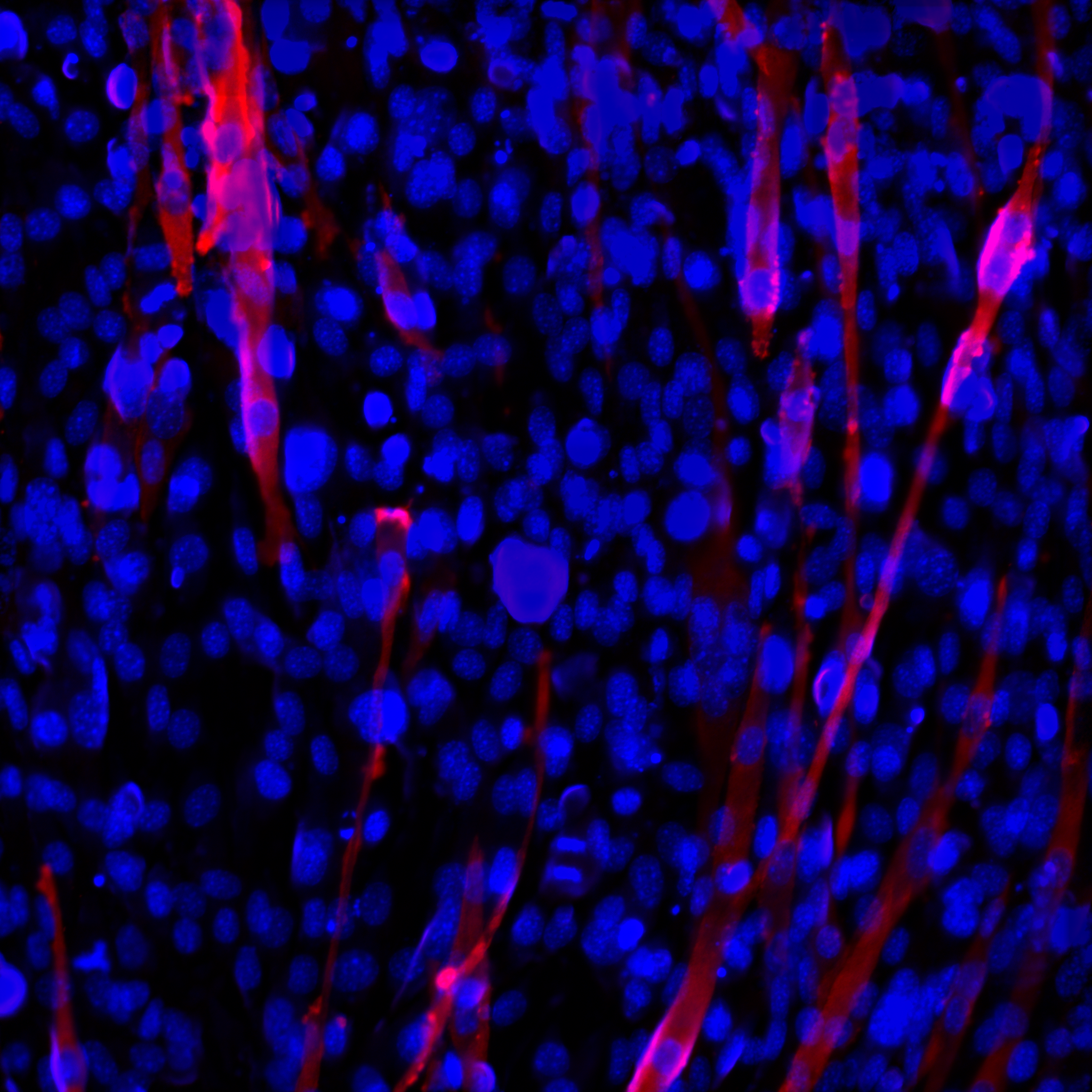

Supplement: S1 File — (ZIP) [file pone.0275298.s004.zip › Supporting Information (images)/Fig5A-04-0mM-HRAS-sTRSVctrl.tif]

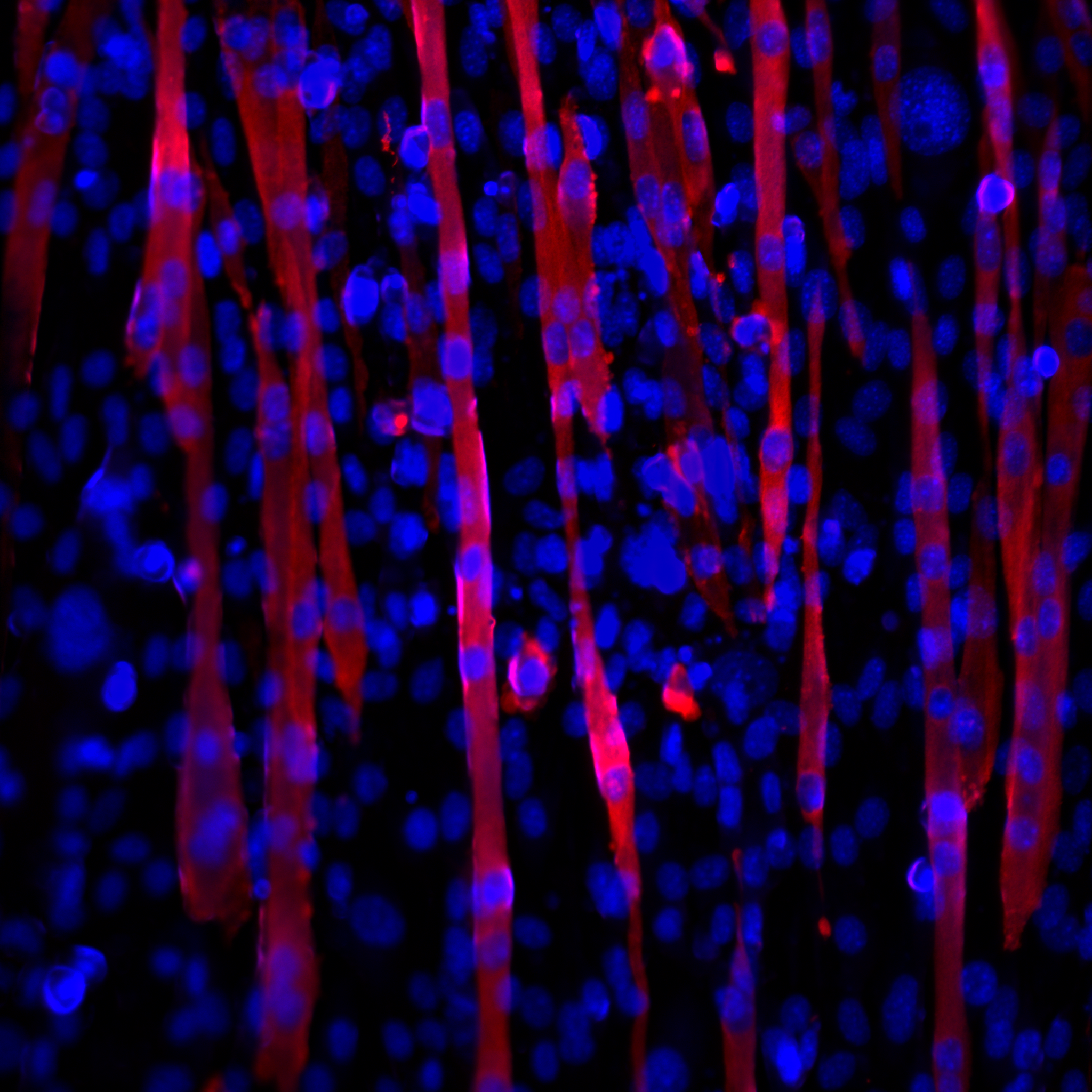

Supplement: S1 File — (ZIP) [file pone.0275298.s004.zip › Supporting Information (images)/Fig5A-02-0mM-HRAS-TheoCAUAA.tif]

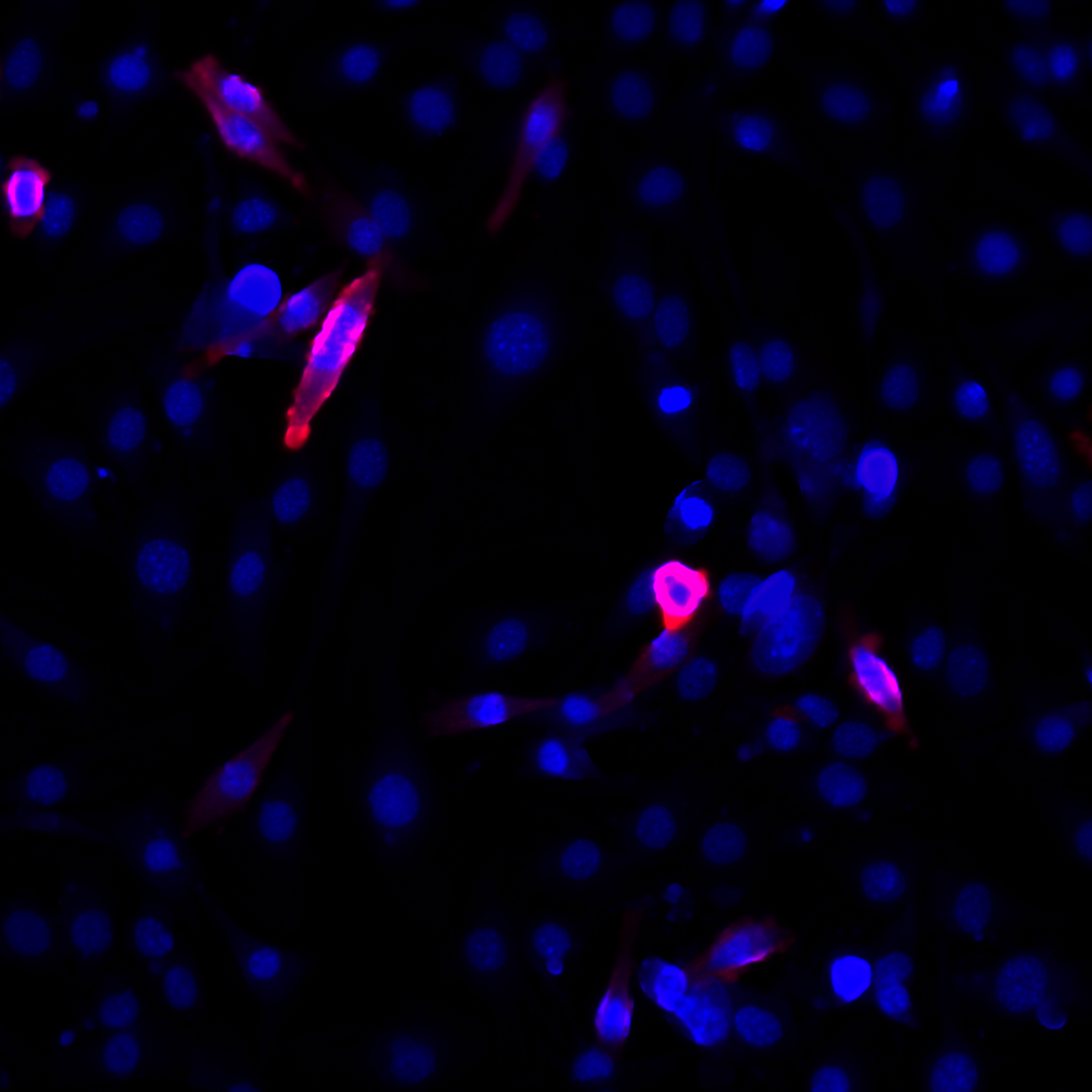

Supplement: S1 File — (ZIP) [file pone.0275298.s004.zip › Supporting Information (images)/Fig5A-06-1mM-HRAS-TheoCAUAA.tif]

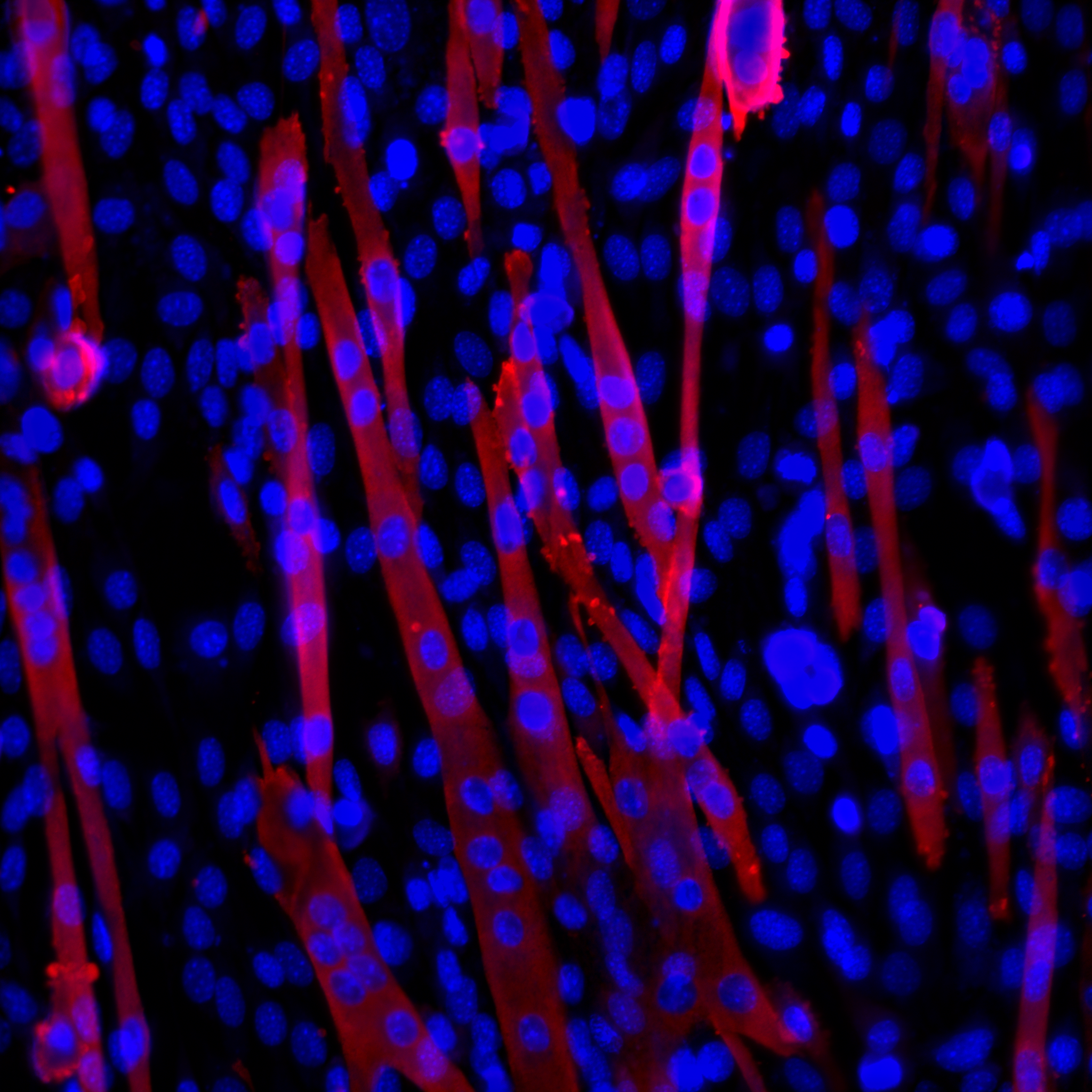

Supplement: S1 File — (ZIP) [file pone.0275298.s004.zip › Supporting Information (images)/Fig5A-01-0mM-HRAS-sTRSV.tif]

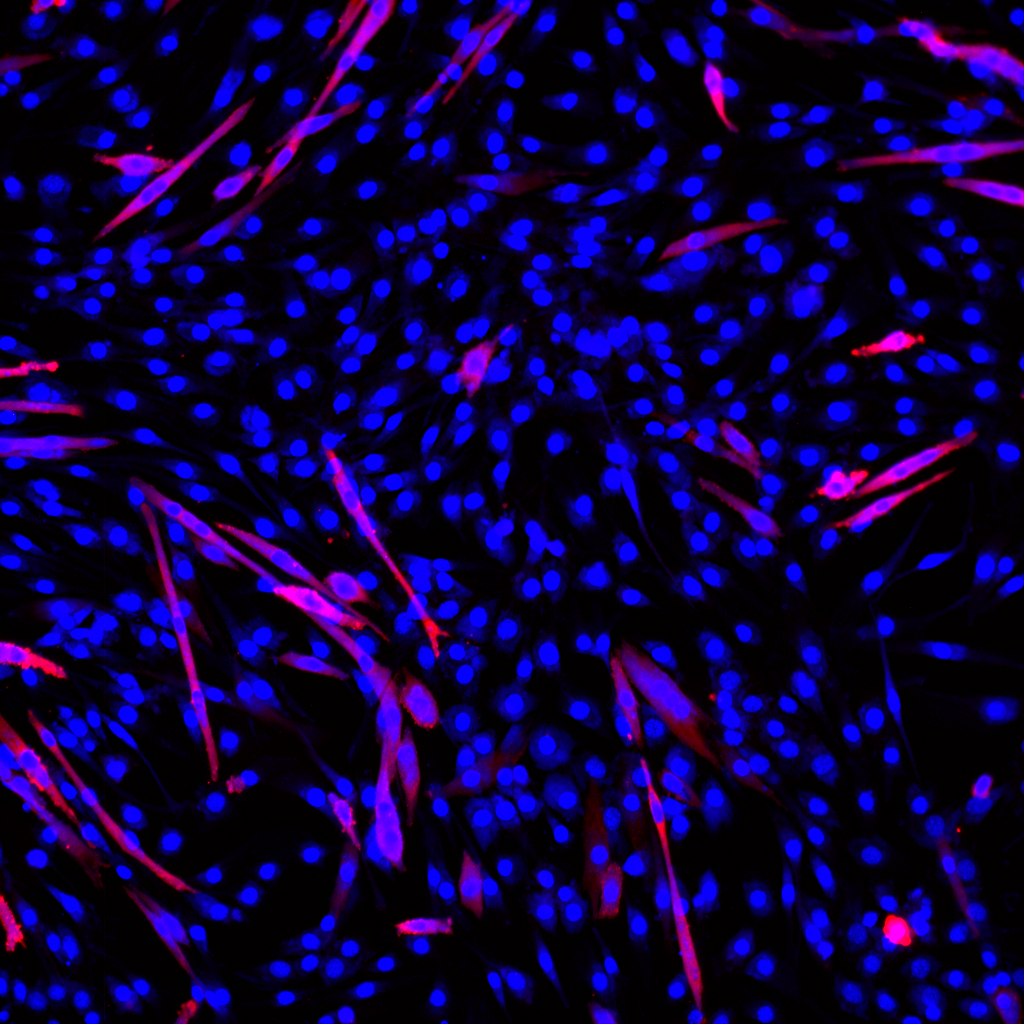

Supplement: S1 File — (ZIP) [file pone.0275298.s004.zip › Supporting Information (images)/Fig3B-03-JAK1.png]

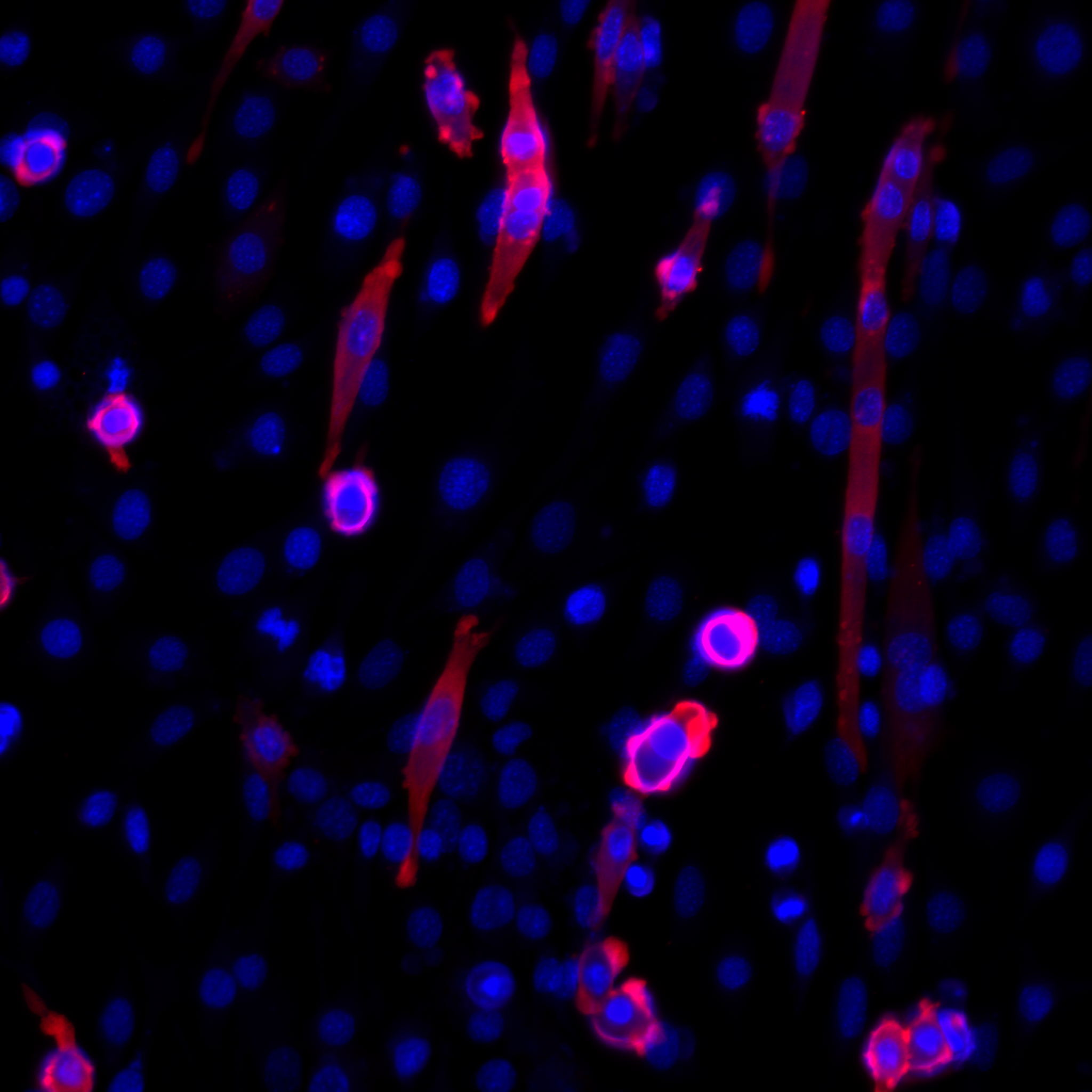

Supplement: S1 File — (ZIP) [file pone.0275298.s004.zip › Supporting Information (images)/Fig5A-05-1mM-HRAS-sTRSV.tif]

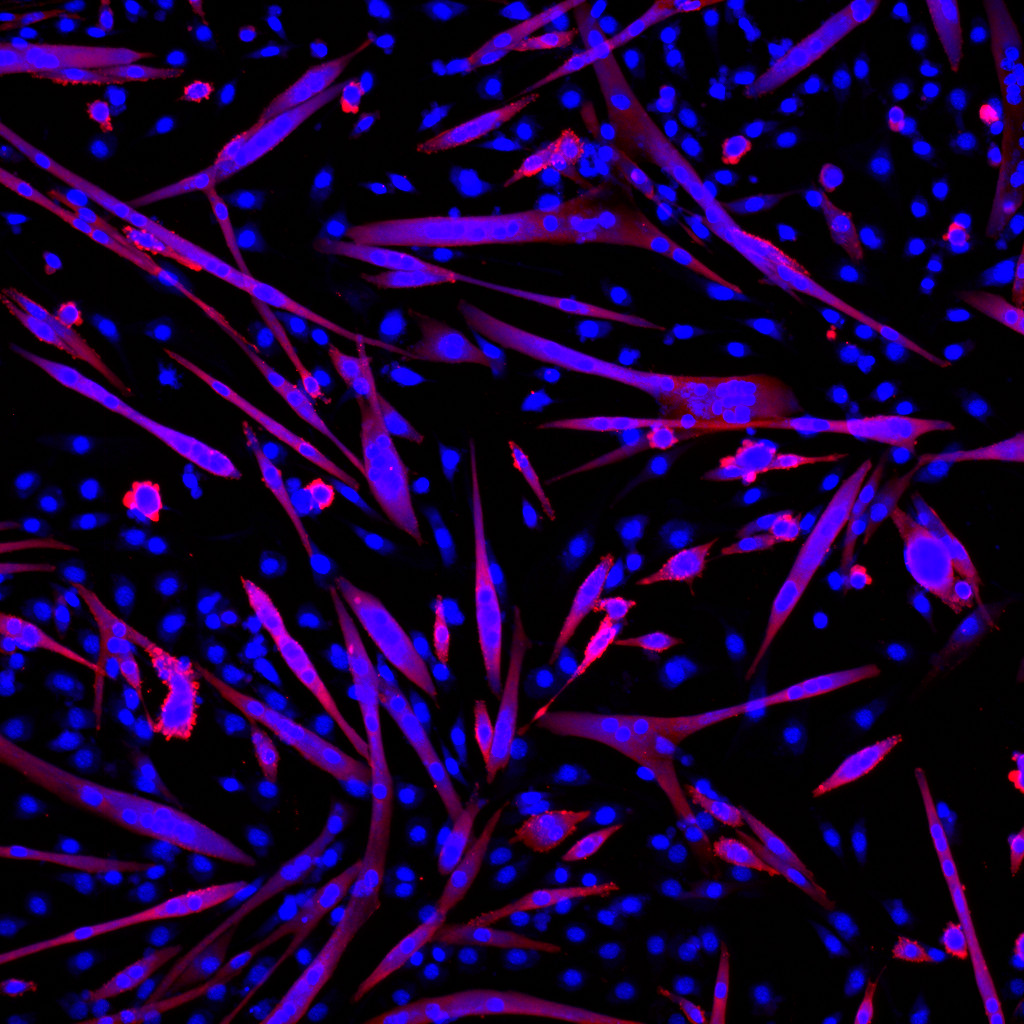

Supplement: S1 File — (ZIP) [file pone.0275298.s004.zip › Supporting Information (images)/Fig3B-01-WT.png]

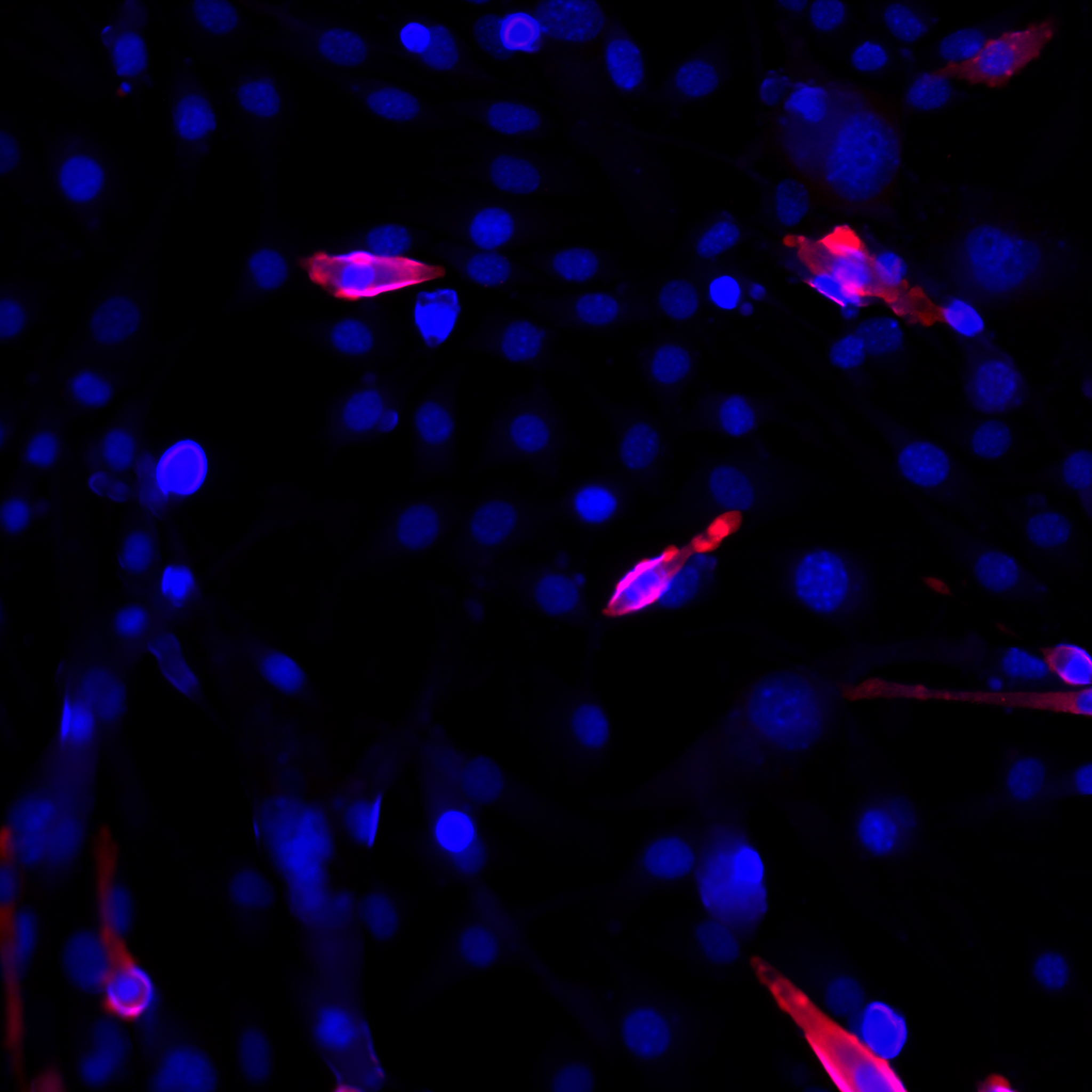

Supplement: S1 File — (ZIP) [file pone.0275298.s004.zip › Supporting Information (images)/Fig5A-07-1mM-HRAS-TheoAGAAA.tif]

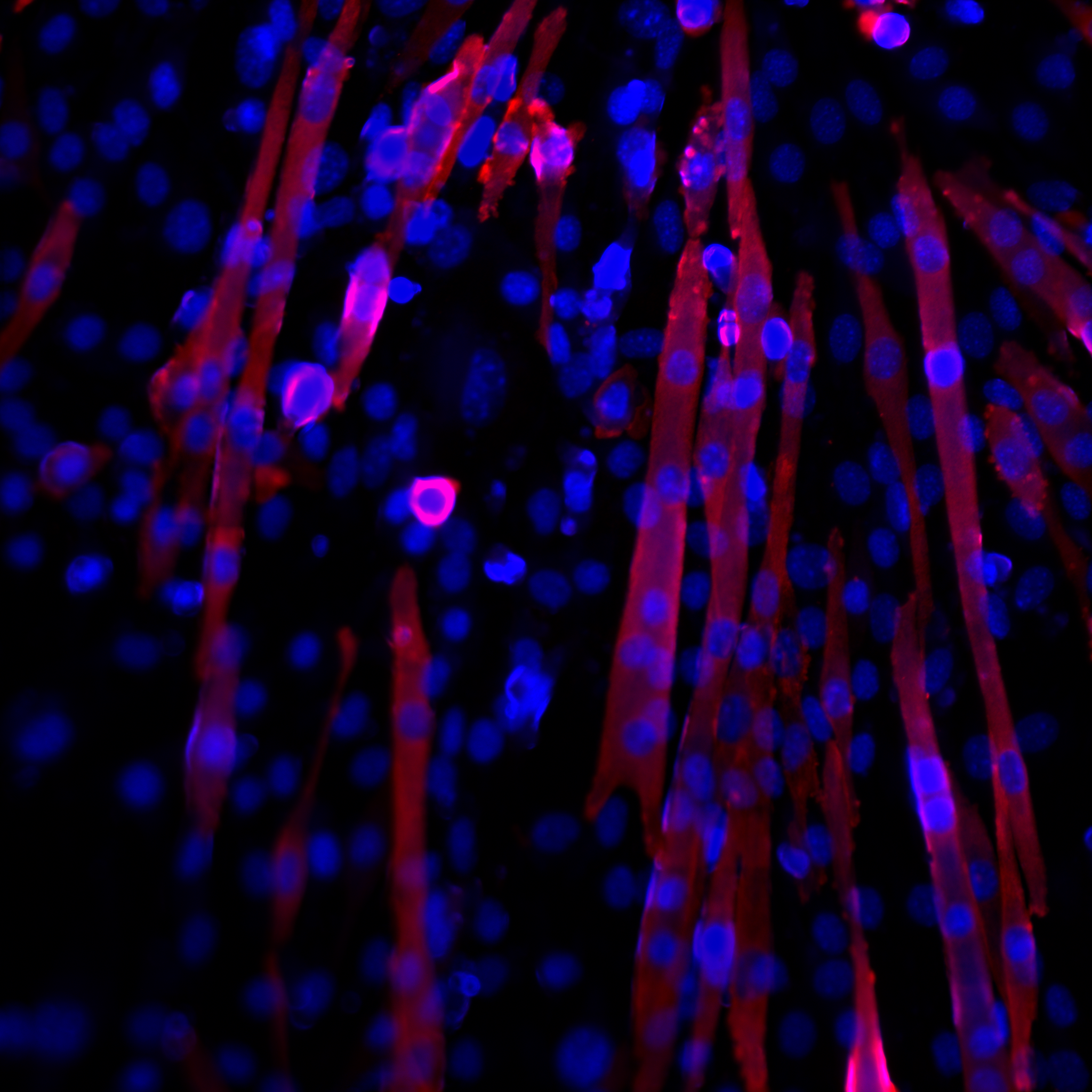

Supplement: S1 File — (ZIP) [file pone.0275298.s004.zip › Supporting Information (images)/Fig5A-03-0mM-HRAS-TheoAGAAA.tif]

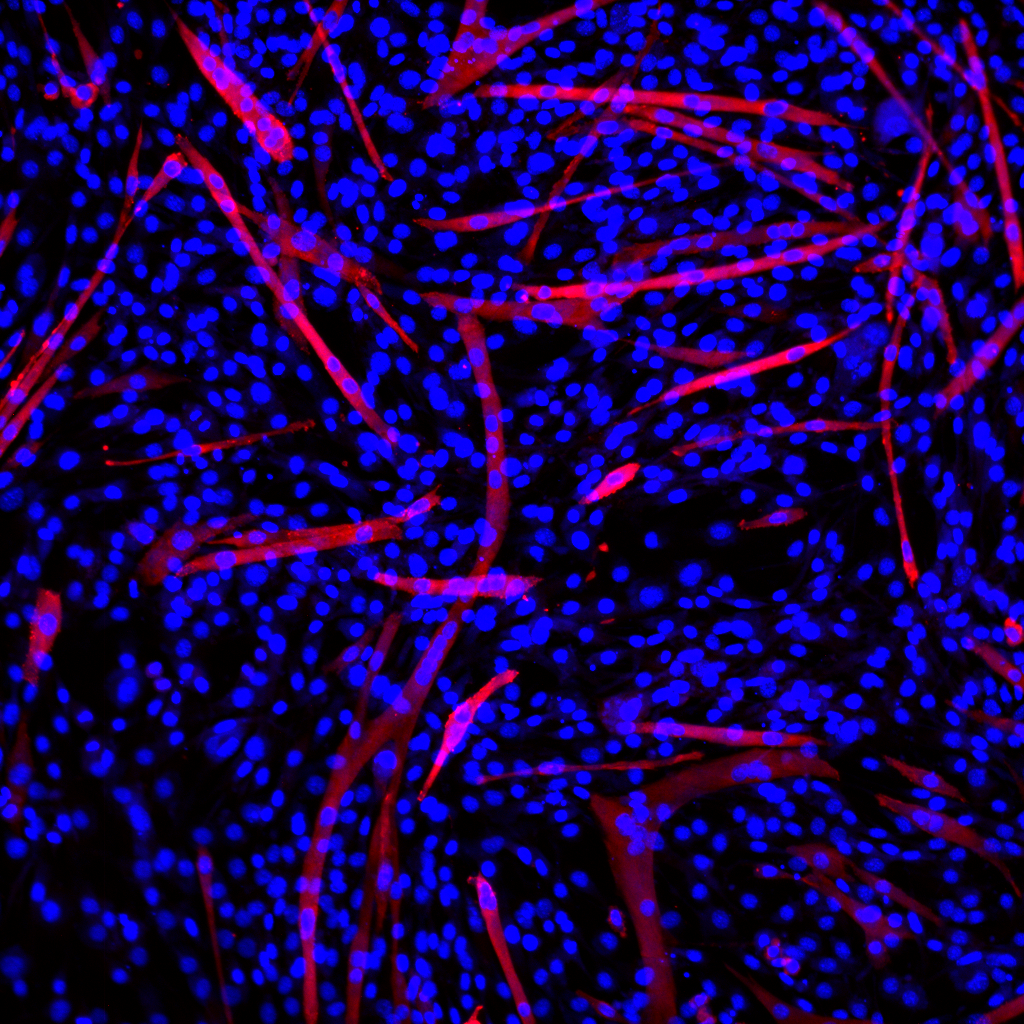

Supplement: S1 File — (ZIP) [file pone.0275298.s004.zip › Supporting Information (images)/Fig3B-02-HRAS.png]
