# Supplementary material for: Modulating myoblast differentiation with RNA-based controllers
Source: PLoS One. 2022 Sep 27;17(9):e0275298. doi: 10.1371/journal.pone.0275298 (PMC9514614; doi:10.1371/journal.pone.0275298)
Supplement: Attachment — Submitted filename: Response to Reviewers.pdf [file pone.0275298.s006.pdf]

Response to Editorial and Reviewer Comments:
We thank the reviewers and editor for their valuable feedback and their constructive comments. Our point-by-point responses to the questions, comments and suggestions provided by the reviewers are provided in italics below.

Journal Requirements:

When submitting your revision, we need you to address these additional requirements.
1. Please ensure that your manuscript meets PLOS ONE's style requirements, including those for file naming.

We have revised the manuscript to meet PLOS ONE's style requirements as requested.

2. In your Data Availability statement, you have not specified where the minimal data set underlying the results described in your manuscript can be found. PLOS defines a study's minimal data set as the underlying data used to reach the conclusions drawn in the manuscript and any additional data required to replicate the reported study findings in their entirety. All PLOS journals require that the minimal data set be made fully available. For more information about our data policy, please see http://journals.plos.org/plosone/s/data-availability.

"Upon re-submitting your revised manuscript, please upload your study's minimal underlying data set as either Supporting Information files or to a stable, public repository and include the relevant URLs, DOIs, or accession numbers within your revised cover letter. For a list of acceptable repositories, please see http://journals.plos.org/plosone/s/data-availability#loc-recommended-repositories. Any potentially identifying patient information must be fully anonymized.

Important: If there are ethical or legal restrictions to sharing your data publicly, please explain these restrictions in detail. Please see our guidelines for more information on what we consider unacceptable restrictions to publicly sharing data: http://journals.plos.org/plosone/s/data-availability#loc-unacceptable-data-access-restrictions. Note that it is not acceptable for the authors to be the sole named individuals responsible for ensuring data access.

We will update your Data Availability statement to reflect the information you provide in your cover letter.

We have included supporting information files to present the minimal data set and all sample image data underlying the results described in the manuscript. We have uploaded the supporting information files and include a description in the cover letter.

Reviewer #1 Comments:
Dykstra et al. characterise two previously described theophylline-responsive ribozyme-based RNA switches in the mouse myoblast C2C12 cell line and use these constructs to control an anti-differentiation signal to allow the expansion of these cells in differentiation inducing media. They use fluorescence reporter to show that both of these theophylline-responsive ribozyme switches are working in the C2C12 cell line as designed (mRNA is degraded/off without theophylline, while stabilised/on with theophylline). The authors then use these constructs to regulate the levels of HRAS or JAK1 mRNAs depending on the presence or absence of

theophylline and show that the HRAS construct can inhibit/slow down myoblast differentiation in a theophylline-dependent manner. Overall, the study is scientifically solid and the conclusions are supported by the data presented in the manuscript.

We thank the reviewer for their positive feedback on the manuscript.

Major comments:
- The authors use the HRAS-sTRSV or Jak1-sTRSV constructs to normalise all myoblast differentiation experiments (Fig 4 C-F and Fig 5), however, this might be misleading if the residual amount of HRAS or Jak1 signal that is still present in these constructs can already slow down differentiation. The fluorescence construct show ~10% signal in the sTRSV construct compared to the non-cleaving construct, while not likely, it is possible that already a low level of HRAS or Jak1 might have an effect on the differentiation of these cells. A simple control showing that the HRAS-sTRSV or Jak1-sTRSV constructs have no effect on the differentiation of these cells would further solidify the conclusions of the manuscript.

We thank the reviewer for this comment. We have added a new supporting figure (Supporting Figure 2) presenting Myog/MyHC transcripts and comparing sTRSV/sTRSVctrl constructs for both HRAS and JAK1 with WT samples. We observe a decrease in Myog and MyHC transcript levels for all constructs, including HRAS-sTRSV and JAK1-sTRSV, relative to WT. Myog transcript levels decreased by 45% for HRAS-sTRSV and 56% for JAK1-sTRSV, relative to WT. MyHC transcript levels decreased by 67% for HRAS-sTRSV and 39% for JAK1-sTRSV, relative to WT. This effect could be related to low levels of HRAS or JAK1 affecting differentiation. However, we believe using sTRSV and sTRSVctrl constructs as negative and positive controls, respectively, enables useful interrogation of the theophylline-responsive devices. Furthermore, these data reinforce our findings regarding the comparative differences between HRAS-sTRSV and HRAS-sTRSVctrl and limited differences between JAK1-sTRSV and JAK1-sTRSVctrl.

- It is a bit unclear what the major conceptual outcome of this study is - beyond that these ligand-responsive ribozyme switches work in these cell line? The idea that these constructs could be used in clinical applications is far-fetched, given the risks associated with transfecting cells with DNA constructs that contain a very strong oncogene. Would it be possible to transfect these constructs as RNA (probably already loaded with theophylline)? If yes, what would be the advanatge compared to just using HRAS or Jak1 mRNAs (without ligand-induced rybozyme switch) to delay differentiation? A short paragraph in the discussion would help the readers to better understand the significance of this study.

We thank the reviewer for this comment and question. The work is presented as a proof-of-concept demonstrating that ligand-responsive ribozyme switches are functional in myoblast cells, building from previous demonstrations in the yeast Saccharomyces cerevisae, HEK cells, and mammalian T cells. For any eventual downstream clinical application, we would envision future research to identify more clinically relevant targets compared to the targets presented here. We have added text to the Discussion Section to clarify this point (lines 362-364 in the final manuscript), and also specifically mention two potential targets beyond the canonical myogenic targets, ALKBH1 and TGFBR1 (lines 359-362).

The specific advantages of these ribozyme-based devices over an mRNA-based strategy focus on the tunable control inherent within the device, as opposed to dosing larger amounts of mRNA. Furthermore, with more clinically relevant targets in the future, the ribozyme-based strategy would offer dynamic control of constructs integrated into the genome via CRISPR/Cas systems. We have added discussion of these topics in the second paragraph of the Discussion Section (lines 321-322, 341-342).

Minor comments:
- Fig 3B – The DAPI signal is barely visible on the Figure and it is very hard to notice that e.g. the nuclei number is increased in the hRAS sample.

We thank the reviewer for this comment. We have increased the contrast of the DAPI signal in a revised Figure 3B to make the signal more visible.

- figure 4 legend : "Values are means normalized to either sTRSVctrl (A and D) or sTRSV (B, C, E, F);" is most likely a typo, A and B are normalized to sTRSVctrl and C-F are normalized to sTRSV

We thank the reviewer for this comment. We have revised this figure legend to remove the typo and state that A and B are normalized to sTRSVctrl while C-F are normalized to sTRSV (line 247).

Reviewer #2 Comments:
The Authors of the manuscript Modulating myoblast differentiation with RNA controllers (Smolke et al) have expended on their initial findings which since have been implemented in several cellular and non cellular models successfully. The present study use the same transfection system to inhibit c2c12 myoblasts into myotubes through response to exogenous theophylline supplement.

Major concerns:
While the general idea/goal of the study is clear and the proof of concept is somewhat obtained as per previous studies by the authors, what is unclear to this reviewer is the choice of the test genes for this specific cell line. C2c12 are robust and adjustable myoblasts used in many proof of concept studies. The myogenic pathway and other pathways dependent and interacting with this pathway are well known, as well as most genes playing part in these pathways. Hence, the logic of why use HRAS which is remotely associated with myogenic differentiation is unclear. Was the hypothesis that this "over arching" gene will have an effect on any differentiating cells. As it turned out to be, transfections with JAK1 which is associated with the myogenic pathway did not result in such a significant ability to switch on/off genes associated with myogenesis. It is of importance to do the same transfection in genes that are downstream and are directly related to the myogenic pathway (Pax7, MyoD etc).

An array of genes specifically related to the myogenic pathway in a stage specific manner can be tested, however, if the end point is fusion and formation of tubes, would the authors consider identifying the expression of genes directly associated with fusion (ie; myomaker etc). Also, it is important to confirm gene expression with protein expression. Since only couple of genes were examined, this experiment should be added.

We thank the reviewer for these comments. Our strategy for choosing test genes included literature review of cell signaling pathways shown to affect myoblast fusion and formation of myotubes as well as the inclusion of an exceedingly strong pro-proliferation signal. We identified JAK1 via the JAK-STAT signaling pathway and tested HRAS alongside as a strong signal. We have added clarifying comments to the manuscript text to highlight why HRAS was selected (lines 168-170).

We agree that future studies should test genes associated with early steps in the myogenic pathway (i.e., Pax7, MyoD) as well as an array of genes directly associated with fusion (including myomaker, myomerger, and others). We believe such follow-up studies are outside the scope of this proof-of-concept paper and will be best addressed in future studies. In the manuscript, we have modified the text in the Discussion Section to highlight these points and potential target genes (lines 362-364).

We acknowledge the reviewer's suggestion for confirmation of protein expression patterns (i.e., Western blot). In the manuscript, we present direct transcript data for the target genes HRAS and JAK1 (Fig 4A-B), indirect transcript data for the downstream genes HRAS and JAK1 would impact (i.e. Myog and MyHC) (Fig 4C-F), and direct phenotypic data for differentiation via the fusion index (Fig 5). Given that these datasets align with the proposed mechanism of the RNA device, we believe that Western blots are not necessary to support the claims of the manuscript as presented. In addition, previous work has shown that regulation of transcript levels via ribozyme-based RNA devices shows strong correlation between resulting transcript levels and protein expression (ref. 20, Xiang et al.).

What is the reason of testing the ribozyme switch in PGK and EF1A first. The method was clearly repeated in the test genes (HRAS and JAK1). Instead a dose response to theophylline and cell viability experiments should be conducted. The authors indicate that there was some toxicity to theophylline, but except of indicating that this occurs above 1 mM concentration and refer to other studies, they should show this clearly in the current study.

The ribozyme switches had previously been characterized in yeast (Saccharomyces cerevisiae), HEK cells, and mammalian T cells, but had not been characterized in muscle myoblasts. Before incorporating effector proteins (i.e., HRAS and JAK1) the ribozyme switches were characterized using flow cytometry to determine if the switches would be active in myoblasts. This initial characterization was performed by evaluating mCherry expression levels driven by the EF1A promoter and controlled by the ribozyme switches. Clover expression driven by the PGK promoter was used as a transfection control. Once performing the initial characterization of the ribozyme switch activities in the myoblast cell line, mCherry was exchanged for either HRAS or JAK1. The final constructs contained HRAS or JAK1 driven by the EF1A promoter.

We also thank the reviewer for their comment and suggestion regarding a theophylline dose response curve and cell viability. We had presented a dose response curve of theophylline-controlled mCherry expression in the myoblast cell line in Figure 2C. We have added a new supporting figure (Supporting Figure 2) showing HRAS transcript levels in response to theophylline levels. We have also added a new supporting figure (Supporting Figure 1) showing cell viability in response to theophylline levels. We observed decreased cell viability at higher doses of theophylline, dropping to around 50% viability at 1 mM theophylline, in line with previously reported findings [see refs. 20 and 29].

The authors indicate on 4 experimental groups (lines 196-200). Those grown in the presence or not of theophylline and then differentiated in the presence or not of theophylline. Which one of these groups are presented in figure 3?. Additionally, will cells grown in the presence of theophylline followed by removal of theophylline in a differentiation medium (transfected with either HRAS or JAK1), form tubes with a normal fusion index as WT?

Only 0 mM theophylline groups are shown in Figure 3. We have revised the text to remove references to 0 mM or 1 mM theophylline as these data are presented prior to the incorporation of theophylline-responsive switches (lines 198-199).

The reviewer also raises an interesting consideration regarding the timing of the theophylline effect. We did not perform the specific experiment the reviewer mentions in their comment (i.e., growing cells in growth medium with theophylline before differentiating in differentiation medium without theophylline). However, we have observed that growing cells transfected with HRAS and JAK1 in growth medium with theophylline shows a general decreasing effect on cell number due to theophylline toxicity (Supporting Figure 1) and myotube fusion is generally known to be dependent on cell density.

The fact that JAK1-based controllers had negative results is an indication that the ribozyme switch may be effective in "master" controller genes such as HRAS acting in an "all or nothing" way such as in cancer and embryonic cells, but not in downstream genes which are more specific to pathways associated with tissue specific differentiation. This means that the method which is ingenious is not yet suitable to switch on/of genes related to tissue specific diseases, and in this case, such as muscle dystrophy.

We note an additional hypothesis to explain the negative results from the JAK1-based controllers. The regulation of JAK1 expression could result in concurrent up- or down-regulation in other redundant genes to preserve the myogenic pathway. We have added this possible interpretation to the manuscript text in the Discussion Section and point out strategies to target downstream genes with a larger effect as well as alternative gene targets to test in future studies (lines 338-339, 356-364).

Minor comments:
All figures: Are the bars indicate SEM or SD?

We thank the reviewer for this question. All bars in the figures indicate standard deviations. This is noted at the end of each figure legend (e.g., lines 155-156, 159-160, 190-191, etc.).

Fig 2B concentrations of theophylline are in M and in 2C in uM. It will be useful to use similar terms as appear in all figures.

We thank the reviewer for this suggestion. We have revised Figure 2C based on the reviewer's suggestion. All theophylline concentrations are now in mM.

Figure 4E and F indicates significance of 0.01 in groups with either SEM or SD (???) which overlap. This needs to be retested. Was the T-test a paired T-Test and was a post-hoc used?.

We thank the reviewer for this comment and clarify that Figures 4E and 4F do not indicate significance between groups and are labeled with "ns" rather than an asterisk. We have updated the figures throughout the manuscript to increase the font size of both asterisks indicating significance and "n.s." indicating no significance (e.g., Lines 156-157, 191-192, 249-250, and 306-307). The t-test used was an unpaired t-test without a post hoc comparison.
